# Surgical Experience of Supratentorial Meningiomas Without Preoperative Embolization in a Low-Income Country: A Retrospective Observational Study

**DOI:** 10.7759/cureus.95221

**Published:** 2025-10-23

**Authors:** Talha Sajid, Umer Farooq, Shahrukh Rizvi, Rabia Saleem, Ismaeel K Iqbal, Muhammad Arslan, Ansarullah Khan, Javed Iqbal, Imran Bajwa, Abdul Majid

**Affiliations:** 1 Neurological Surgery, Punjab Institute of Neurosciences, Lahore, PAK; 2 Neurosurgery, PunJab Institute of Neurosciences, Lahore, PAK

**Keywords:** embolization, meningioma, neurosurgical procedures, pakistan, supratentorial neoplasms, therapeutic

## Abstract

Objective: To evaluate the outcomes of supratentorial meningioma resection performed without preoperative embolization at a high-volume neurosurgical public center in Pakistan.

Methods: This retrospective observational study reviewed 100 consecutive patients who underwent surgical excision of supratentorial meningiomas between January 2022 and December 2024 at the Punjab Institute of Neurosciences, Lahore. Patients aged 15 years or older with radiological and histopathological confirmation were included; infratentorial tumors and biopsy-only cases were excluded. Clinical, imaging, and operative data were analyzed. Mean ± standard deviation (SD) values were calculated for operative time, anesthesia time, and estimated blood loss. Pearson correlation analysis was applied to evaluate the relationship between operative duration and blood loss. Extent of resection, perioperative complications, and functional outcomes at discharge were also assessed using the Karnofsky Performance Score (KPS) and Glasgow Outcome Scale (GOS). Patients were followed clinically and radiologically for a median duration of one month (range: two weeks to three months) after surgery to record early postoperative outcomes.

Results: The cohort had a mean age of 46.3 years, with a female predominance of 69 (69%). Headache was observed in 65 (65%) patients, and seizures in 40 (40%). Gross total resection was achieved in 95 (95%) cases, and near-total resection in 5 (5%). The mean operative time was 193 ± 22 minutes, anesthesia time 220 ± 25 minutes, and estimated blood loss 686 ± 145 mL. A moderate positive correlation was observed between operative time and blood loss (r = 0.56). Early postoperative complications included new motor deficits, hydrocephalus, cerebrospinal fluid leak, and shunt requirement, with low in-hospital mortality. Most patients demonstrated favorable early functional recovery (KPS >80, GOS 5).

Conclusion: Supratentorial meningioma surgery without preoperative embolization can achieve favorable early outcomes when performed with disciplined microsurgical technique, controlled hemostasis, and coordinated perioperative care. The findings emphasize that safe maximal resection is feasible even in resource-limited settings and support the need for future multicenter comparative studies, including embolized cohorts.

## Introduction

Meningiomas constitute a major share of primary intracranial tumors in adults; they originate from arachnoid cap cells and typically show a marked female predominance, while their clinical course ranges from indolent to more aggressive patterns that demand tailored follow-up and treatment decisions [1,2]. Across contemporary summaries, the usual pathway includes symptom-guided evaluation, MRI for characterization and surveillance, and WHO grading to estimate risk; in this framework, surgery remains the mainstay for symptomatic or enlarging lesions, with radiotherapy used selectively when anatomy, residual disease, or histology warrants it [3,4]. For supratentorial meningiomas, operative planning emphasizes safe but maximal resection with preservation of eloquent cortex and critical venous structures, and the classic Simpson grading has been reinterpreted in modern practice to account for biological behavior, reconstruction needs, and real-world surgical limits [5,6]. Seizures are common in this subgroup and, together with peritumoral edema, shape perioperative choices such as steroid use, antiepileptic prophylaxis, anesthetic strategy, and early postoperative monitoring aimed at reducing complications and expediting recovery [7,8]. Where available, preoperative embolization of selected hypervascular meningiomas may help decrease intraoperative blood loss and facilitate dissection when performed by experienced teams [9,10]. Recent reports also stress judicious case selection with careful angiographic mapping to avoid ischemic complications [8,9], and a contemporary classification based on embolic-agent penetration has been proposed to standardize reporting and anticipate hemostatic benefit [10]. Rockhill et al. review intracranial meningiomas and affirm that surgical resection remains the cornerstone of management in most patients, while radiotherapy is deployed selectively for residual or recurrent lesions [11].

In Pakistan’s public and many private hospitals, resources are constrained, and neurointerventional capacity remains uneven. Because preoperative embolization is not part of standard meningioma management and is used only in selected cases, even in high-income settings, its use is further limited in our environment by financial, logistical, and expertise constraints [4]. As a result, most neurosurgical teams proceed directly to tumor resection, relying on disciplined microsurgical hemostasis and practical, locally adaptable protocols for edema control, wound management, and seizure prevention that can be delivered with available resources [5].

Despite this being the common surgical pathway, there is very little published data describing outcomes from centers where embolization is not routinely performed. Documenting such experiences can help show how safe and effective surgery can still be achieved through careful technique and teamwork, even when advanced adjuncts are unavailable. Therefore, this study aimed to evaluate the outcomes of supratentorial meningioma resection performed without preoperative embolization at a high-volume public neurosurgical center in Pakistan.

## Materials and methods

Study design and setting

This was a retrospective, single-center observational study conducted in the Department of Neurosurgery, Punjab Institute of Neurosciences (PINS), Lahore, Pakistan. Data abstraction was performed from January 2025 to March 2025 and included all eligible supratentorial meningioma resections carried out between January 1, 2022, and December 31, 2024. A non-probability convenience sampling strategy was used, enrolling all consecutive patients who met the eligibility criteria during the study period. Preoperative tumor embolization was not part of routine care due to limited availability and cost constraints in our setting.

Eligibility criteria are defined in Table 1. 

**Table 1:** Eligibility criteria for study participants

Inclusion Criteria	Exclusion Criteria
Patients ≥15 years of age	Infratentorial meningiomas
Either sex (male or female)	Biopsy-only cases without definitive tumor resection
Histopathological confirmation of supratentorial meningioma	Incomplete medical records

Data collection

Data were retrieved from hospital medical records, operative and anesthesia notes, imaging reports, and the Picture Archiving and Communication System (PACS) and were entered into a structured proforma. Variables included demographics and comorbidities; clinical presentation, symptom duration, and preoperative status (Glasgow Coma Scale and Karnofsky Performance Score [KPS]); imaging location; operative details (surgical approach, operative time, anesthesia time, and estimated blood loss); and postoperative outcomes (new motor deficit, hydrocephalus, cerebrospinal fluid leak, ventriculoperitoneal shunt, KPS at discharge, Glasgow Outcome Scale [GOS], and in-hospital mortality). Extent of resection (EOR) was defined in advance using the Simpson grading system: gross total resection (GTR) = Simpson grades I-II; near total resection (NTR) = Simpson grade III. Complication categories were treated as mutually exclusive, with each patient counted once. Tumor size and histopathological grade variables were not included, as these parameters were not part of the initial data collection framework. 

Postoperative follow-up was conducted through inpatient assessment and outpatient visits within three months after surgery. Clinical evaluations included neurological examination and functional status scoring, while imaging follow-up consisted of postoperative CT scans; MRI was obtained only when clinically indicated. Outcomes recorded during this period were categorized as early postoperative results.

Surgical technique

All surgeries were performed under general anesthesia with endotracheal intubation and standard intraoperative monitoring. Controlled hypotension was maintained throughout tumor resection to minimize intraoperative blood loss. Hemostasis was achieved using bipolar cautery, meticulous irrigation, and hemostatic agents as required. Tranexamic acid was administered intravenously in all cases for antifibrinolytic support. The surgical approach emphasized early identification and devascularization of the tumor capsule prior to bulk excision, which facilitated dissection and reduced intraoperative bleeding. Closure was performed in layers with watertight dural repair, and standard postoperative wound care protocols were followed.

Operations were conducted by multiple neurosurgical teams within the same department, following uniform institutional protocols for preoperative optimization, intraoperative management, and postoperative care to minimize inter-team variability.

Functional status and outcome measures

Functional status at discharge was assessed using the KPS and the original 5-point GOS. Both are widely used and publicly available clinical tools that do not require licensing for academic use. The KPS, originally described by Karnofsky et al. [12], is indicated for evaluating a patient’s ability to perform daily activities and overall functional independence, and it has been extensively used in oncology, neurosurgery, and palliative care to assess baseline status and postoperative recovery. The GOS, developed by Jennett and Bond [13], was primarily designed to evaluate global neurological outcome following traumatic brain injury, but it has since been applied in other neurosurgical contexts, including brain tumor surgery. Tay MRJ et al. [14] used GOS to assess disability and functional recovery in patients with primary brain tumors. In this study, both KPS and GOS were applied by treating teams at the time of hospital discharge to provide a standardized measure of functional outcomes.

Statistical analysis

Data analysis was performed using Microsoft Excel (Microsoft Corp., Redmond, USA), employing descriptive statistics for continuous variables (mean, range, and standard deviation) and frequency distributions for categorical variables. No inferential statistical comparisons between meningioma subtypes were conducted, as the study objective was limited to descriptive evaluation of early intraoperative and postoperative outcomes. To explore potential relationships between intraoperative parameters, a Pearson correlation analysis was conducted between operative time and estimated blood loss. The Pearson correlation coefficient (r) was calculated to assess the strength and direction of the linear association between these continuous variables. A positive r value indicated a direct relationship, whereas a negative value denoted an inverse relationship.

Ethical approval

The study complied with institutional and national guidelines and the Declaration of Helsinki. The institutional review board granted an exemption for this retrospective analysis (IRB Exemption #2098/IRB/PINS/Approval/2025), and the requirement for individual informed consent was waived due to the use of de-identified data and minimal risk to the participants.

## Results

The mean age was 46.3 ± 13.8 years; 69% (n = 69) were female and 31% (n = 31) were male. Comorbidities included hypertension in 13% (n = 13), diabetes mellitus in 9% (n = 9), and neurofibromatosis type 2 in 3% (n = 3).

The most frequent presenting symptom was headache in 65% (n = 65), followed by seizures in 40% (n = 40), vomiting in 28% (n = 28), hemiparesis in 22% (n = 22), visual disturbances in 17% (n = 17), anosmia in 7% (n = 7), and urinary incontinence in 2% (n = 2). At presentation, the Glasgow Coma Scale was 15 in 94% (n = 94), 14 in 4% (n = 4), 10 in 1% (n = 1), and 7 in 1% (n = 1). Preoperative KPS ≥80 in 84% (n = 8). An example of a frontal parafalcine meningioma with peri-lesional edema, showing preoperative MRI and postoperative CT with persistent postoperative edema, and was below 80 in 16% (n = 16). Symptom duration was less than one month in 28% (n = 28), 1-6 months in 46% (n = 46), 6-12 months in 8% (n = 8), and more than 12 months in 18% (n = 18).

**Figure 1:**
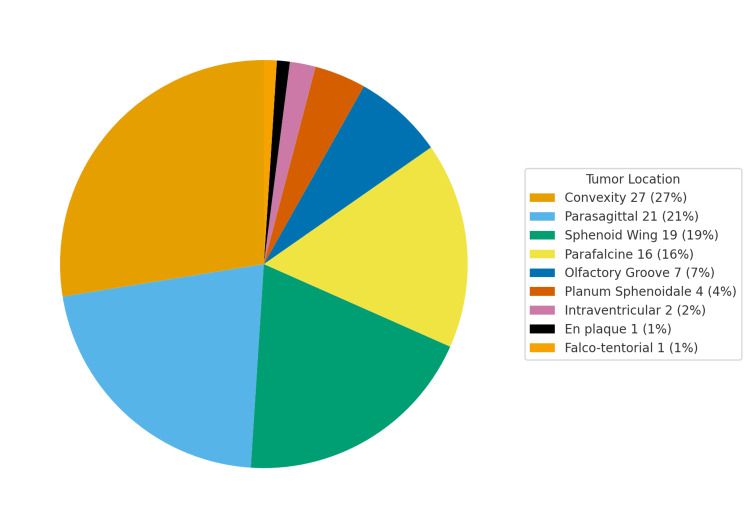
Distribution of supratentorial meningioma locations in this cohort (n=100). Convexity (27%), parasagittal (21%), sphenoid wing (19%), parafalcine (16%), olfactory groove (7%), planum sphenoidale (4%), intraventricular (2%), en plaque (1%), and falco-tentorial (1%) meningiomas. The image is created by the author.

Tumor location distribution is shown in Figure 1; convexity meningiomas were most common, followed by parasagittal, parafalcine, and sphenoid wing sites. 

All patients underwent resection. Gross-total resection, defined as Simpson grades I-II, was achieved in 95% (n = 95); near-total resection, defined as Simpson grade III, in 5% (n = 5). The distribution of Simpson grades was I in 31% (n = 31), II in 64% (n = 64), and III in 5% (n = 5). 

Operative metrics

The mean operative time was 193 ± 22 minutes, and the mean anesthesia time was 220 ± 25 minutes. The mean estimated intraoperative blood loss was 686 ± 145 mL. A Pearson correlation analysis demonstrated a moderate positive relationship between operative time and estimated blood loss (r = 0.56), indicating that longer operative durations were associated with higher intraoperative bleeding.

**Figure 2:**
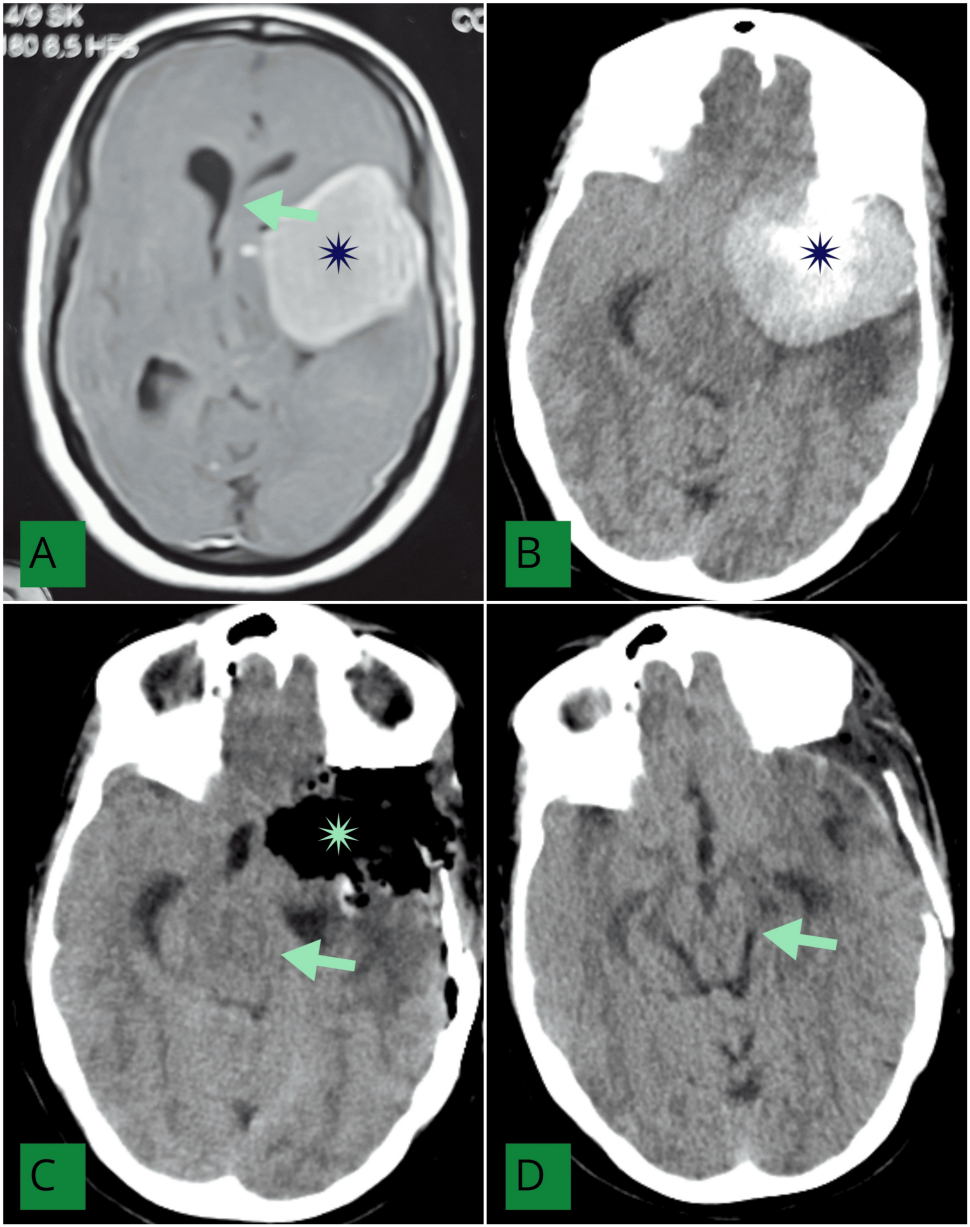
(A) Post-contrast MRI brain (axial view) showing an extra-axial, homogeneously, and densely enhancing lesion in the left frontotemporal region (asterisk) with associated midline shift (green arrow). (B) Preoperative non-contrast CT scan (axial view) demonstrating a hyperdense lesion arising from the left sphenoid wing, displacing the brainstem contralaterally (asterisk). (C) Immediate postoperative non-contrast CT scan (axial view) showing complete resection of the lesion (Simpson grade I) (asterisk). The midline shift persists in the immediate postoperative period (green arrow). (D) One-month postoperative CT scan showing resolution of the midline shift (green arrow). Postoperative CT scan at one-month follow-up demonstrating minor bone-flap malalignment without clinical consequences. The patient had an uneventful recovery. MRI: Magnetic resonance imaging, CT: Computed tomography

Representative imaging of a left frontotemporal meningioma with associated mass effect is shown in Figure 2, including preoperative MRI, preoperative and postoperative CT scans, and resolution of midline shift at follow-up.

**Figure 3:**
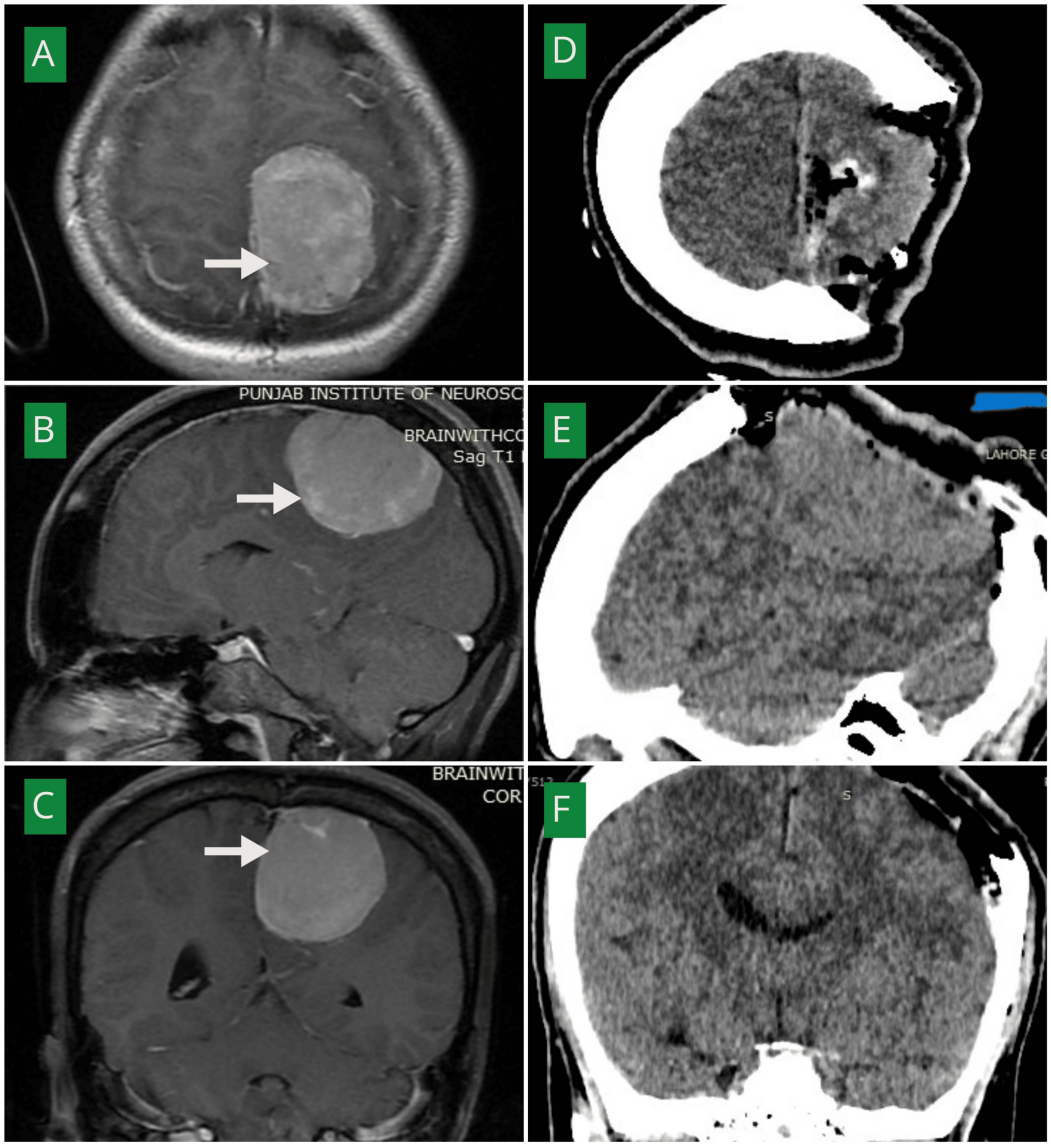
(A–C) Post-contrast MRI brain axial (A), sagittal (B), and coronal (C) views demonstrating a homogeneously enhancing extra-axial mass along the left parafalcine region (white arrows). (D–F) Post-operative non-contrast CT brain axial (D), sagittal (E), and coronal (F) views showing Simpson grade I excision. Postoperative CT scan showing craniectomy performed for tumor excision. The bone was not replaced at the time of surgery due to intraoperative bone involvement and concern for postoperative cerebral edema. Delayed cranioplasty was planned for this patient. MRI: Magnetic resonance imaging, CT: Computed tomography

Figure 3 demonstrates a parafalcine meningioma with preoperative MRI and postoperative CT findings, illustrating gross total excision without peri-lesional infarction.

**Figure 4:**
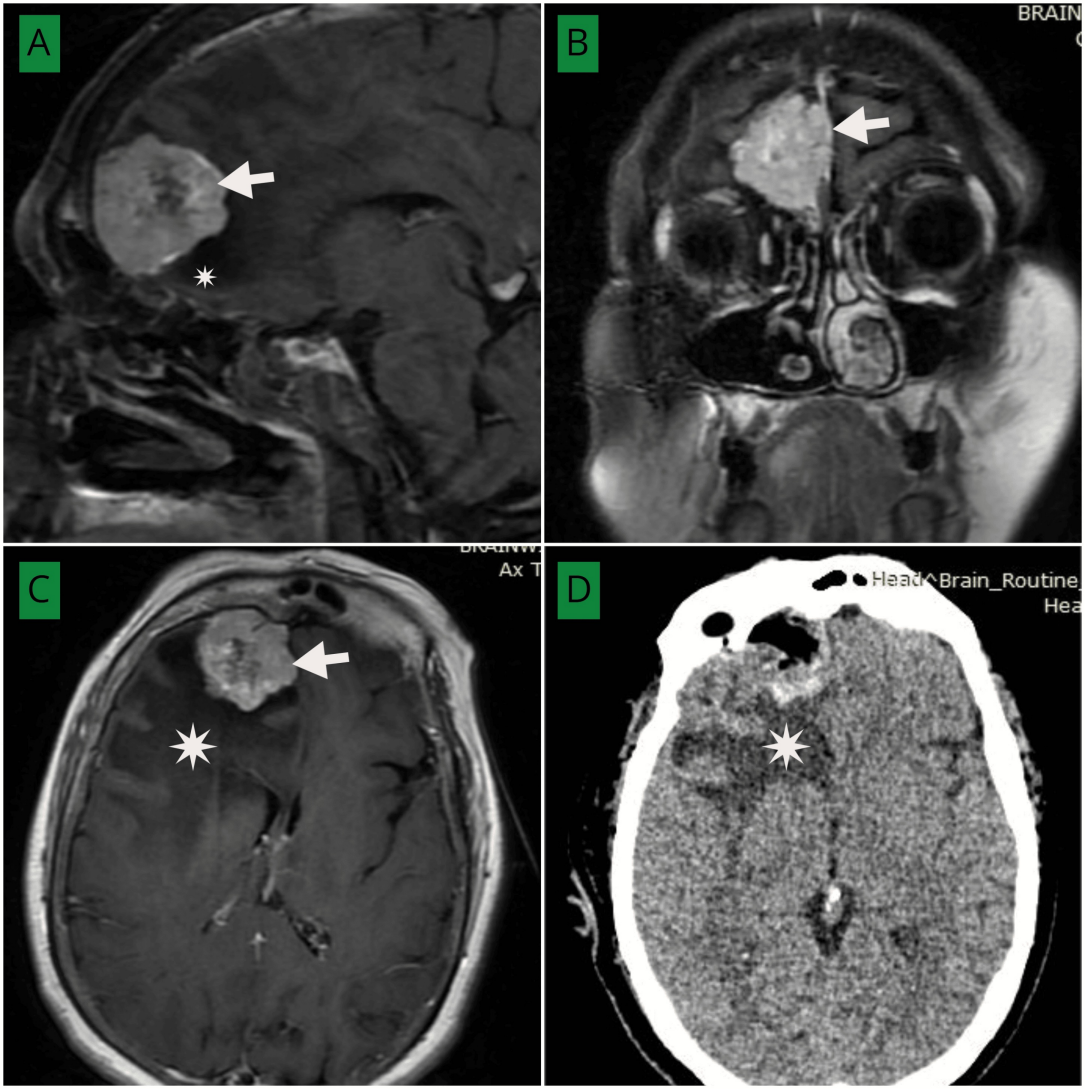
(A–C) Post-contrast MRI brain axial (C), sagittal (A), and coronal (B) views demonstrating a homogeneously enhancing extra-axial mass in the right frontal parafalcine region (white arrows) with perilesional edema (white asterisk). (D) Postoperative non-contrast CT brain (axial view) showing complete excision of the tumor with persistent perilesional edema (white asterisk). MRI: Magnetic resonance imaging, CT: Computed tomography

An example of a frontal parafalcine meningioma with peri-lesional edema is illustrated in Figure 4, showing preoperative MRI and postoperative CT with persistent postoperative edema.

**Figure 5:**
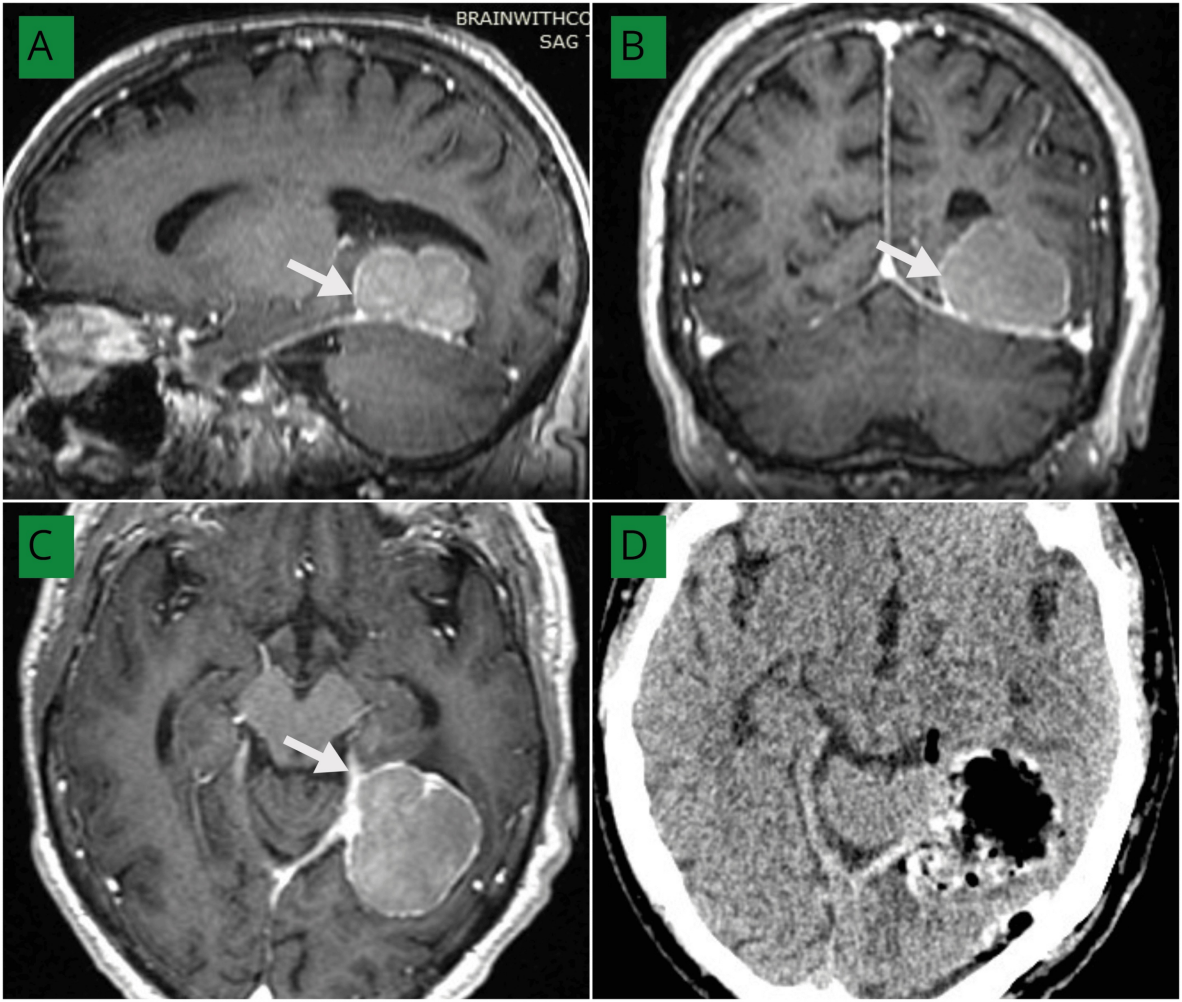
(A–C) Post-contrast MRI brain—axial (C), sagittal (A), and coronal (B) views demonstrating a homogeneously enhancing extra-axial mass attached to the left tentorium cerebelli and extending superiorly (white arrows). (D) Postoperative non-contrast CT brain (axial view) showing complete excision of the tumor. MRI: Magnetic resonance imaging, CT: Computed tomography

Figure 5 depicts a tentorial meningioma, with preoperative MRI and postoperative CT scans confirming complete excision of the lesion.

The median follow-up duration was one month (range: two weeks to three months), during which all early postoperative complications and functional outcomes were recorded.

Early postoperative outcomes included new motor deficit in 7% (n = 7), hydrocephalus in 3% (n = 3), cerebrospinal fluid leak in 2% (n = 2), and ventriculoperitoneal shunt placement in 2% (n = 2); in-hospital mortality was 1% (n = 1). Complication categories were mutually exclusive; each patient was counted in only one complication category. At discharge, KPS was >80 in 87% (n = 87), <80 in 12% (n = 12), and 0 in 1% (n = 1; in-hospital mortality). The GOS at discharge was 5 in 87% (n = 87), 4 in 12% (n = 12), and 1 in 1% (n = 1). This distribution shows expected concordance between KPS and GOS (KPS >80 corresponded to GOS 5; KPS <80 corresponded to GOS 4; death corresponded to GOS 1). The detailed outcomes are given in Table 2. 

**Table 2:** Surgical and postoperative outcomes (n = 100) All values are n (%) unless stated. Complication categories were mutually exclusive (each patient counted once). EOR was defined a priori from Simpson grade: GTR = Simpson I–II; NTR = Simpson III. The death is counted as KPS 0 and GOS 1. KPS: Karnofsky Performance Score; GOS: Glasgow Outcome Scale; CSF: Cerebrospinal fluid; VP: Ventriculoperitoneal. Outcomes were recorded during early postoperative follow-up (median one month, range two weeks–three months).

Parameter	Category / Unit	Value (n, %)
Extent of resection (EOR)	Gross-total (Simpson I–II)	95 (95)
	Near-total (Simpson III)	5 (5)
Simpson grade	I	31 (31)
	II	64 (64)
	III	5 (5)
Operative time	minutes, mean ± SD	193 ± 22
Anesthesia time	minutes, mean ± SD	220 ± 25
Estimated blood loss	mL, mean ± SD	686.2 ± 145
Complications	Motor deficit	7 (7)
	Hydrocephalus	3 (3)
	CSF leak	2 (2)
	VP shunt placement	2 (2)
Mortality	In-hospital	1 (1)
KPS at discharge	>80	87 (87)
	<80	12 (12)
	0 (death)	1 (1)
GOS at discharge	5	87 (87)
	4	12 (12)
	1	1 (1)

## Discussion

This single-center series from a low-income setting adds practical context to supratentorial meningioma care when preoperative embolization is not routinely available. The mean age in our cohort (46.3 ± 13.8 years) was younger than many summaries that emphasize later middle age, while the female predominance (69%, n=69) matched global patterns reported by Ogasawara et al. and Buerki et al. [1,2]. Headache (65%, n=65) and seizures (40%, n=40) were the leading symptoms, in line with broad reviews and seizure-focused cohorts of supratentorial tumors described by Schneider et al. and Chen et al. [1,2,7,15].

Preoperative function was high (KPS > 80 in 84%, n=84), which typically predicts a better early recovery after surgery, as highlighted in contemporary overviews [1,2]. Using Simpson grade to define the extent of resection (EOR), we achieved gross-total resection (GTR; Simpson I-II) in 95% (n=95) and near-total resection (NTR; Simpson III) in 5% (n=5). These rates are compatible with non-skull base supratentorial management, where careful respect for venous anatomy and eloquent cortex supports extensive resection, as outlined by Adekanmbi et al. [5]. The prognostic relevance of the extent of resection remains strong even as the field refines Simpson-based concepts; this perspective is reflected by Dadario et al. and Simon et al. [6,16].

In our cohort, the mean operative time (193 minutes) and anesthesia time (220 minutes) were shorter than those reported in other supratentorial series (~224-255 minutes) [16], whereas the mean estimated blood loss (686.2 mL) was higher than both non-embolized (315 mL) and embolized cohorts (262-410 mL) [10]. This likely reflects the absence of preoperative embolization in our cases and the predominance of large, edematous convexity and parasagittal tumors, consistent with the edema-size relationship described by Shin et al. [17]. When cranial bone involvement is present, operative planning and reconstruction become more complex and can influence both blood loss and operative duration, as outlined by Clynch et al. [18]. The observed moderate positive correlation (r = 0.56) between operative time and estimated blood loss highlights a predictable relationship, as more extensive or technically demanding procedures typically involve greater vascularity and prolonged dissection. Similar associations have been reported by Raper et al. and Yoon et al., where non-embolized meningioma cohorts demonstrated higher intraoperative blood loss proportional to surgical duration and tumor complexity [8,9]. When embolization is available, Raper et al. [9], Yoon et al. [8], and Gutiérrez-Baños et al. [10] have documented significant reductions in intraoperative bleeding and operative difficulty with selective use and meticulous technique. Our findings suggest that disciplined microsurgical hemostasis, early tumor capsule devascularization, and coordinated anesthesia can achieve acceptable blood loss even in the absence of embolization, though the endovascular option remains desirable when institutional resources permit. The comparison is summarized in Table 3. Beyond open resection, magnetic resonance-guided laser interstitial thermal therapy (LITT) provides a minimally invasive alternative for selected recurrences, though accessibility remains limited in low-income settings [19]. 

**Table 3:** Comparison of present study outcomes with published embolized meningioma series.

Study	Embolization Used	Mean Blood Loss (mL)	Gross-Total Resection (GTR, %)	In-Hospital Mortality (%)
Yoon et al., 2018 [8]	Yes	262–410	90–95	< 1
Raper et al., 2014 [9]	Yes	315	93	0.8
Gutiérrez-Baños et al., 2025 [10]	Yes	320	91	0.9
Present Study (2025)	No	686	95	1.0

Early postoperative complications were low: new motor deficit 7% (n=7), hydrocephalus 3% (n=3), cerebrospinal fluid (CSF) leak 2% (n=2), VP shunt 2% (n=2), and in-hospital mortality 1% (n=1). CSF-related events vary with location and the extent of dural work; Chotai et al. reported differences in leak profiles by reconstruction technique, which helps explain the low leak rate in our predominantly non-skull base cohort [20]. Wound complications can add cost and delay recovery in resource-limited hospitals; continued attention to prevention is warranted, as summarized by Wang et al. [21]. At discharge, functional outcomes were favorable and internally consistent (KPS > 80 in 87%, n=87, with concordant GOS 5 in 87%, n=87), echoing the established link between preoperative status, EOR, and early outcomes discussed by Simon et al. [6].

A setting-specific issue is the absence of routine preoperative embolization and selective adjuvant radiotherapy. Current guidance supports considering embolization for hypervascular tumors in experienced centers and postoperative radiotherapy for residual or higher-risk disease; our results show that standard microsurgical pathways can still deliver strong early outcomes when those adjuncts are unavailable, but access remains a meaningful equity gap in low-income environments [4,8-10].

Limitations

This study has several limitations. It was a retrospective, single-center analysis, which may introduce selection and documentation bias and limit the generalizability of the findings. Long-term follow-up data were unavailable, preventing assessment of tumor recurrence, survival, or delayed complications. The follow-up period in this series was short, reflecting an early-outcome focus and the practical limitations of postoperative patient tracking in our setting. Tumor size and volumetric measurements, as well as histopathological grades, were not collected as part of the initial dataset, restricting analysis of how these variables may have influenced intraoperative parameters and outcomes. Data on adjuvant therapy, transfusion rates, and cost were also not recorded. Routine postoperative MRI was not feasible in this resource-limited setting and was reserved for patients with new or recurrent symptoms or when CT findings raised concern for residual disease. Furthermore, the absence of a comparative embolized cohort limits inferential interpretation. Despite these constraints, the study provides practical early-outcome data relevant to neurosurgical centers operating in resource-limited environments and highlights areas for further prospective research.

## Conclusions

Supratentorial meningioma surgery performed without routine preoperative embolization can achieve favorable early outcomes when guided by standardized microsurgical technique, controlled hemostasis, and coordinated perioperative care. The addition of correlation analysis and comparative evaluation with embolized cohorts highlights the importance of surgical discipline in resource-limited settings. While these findings reinforce the feasibility of safe maximal resection without embolization, future prospective and multicenter studies comparing embolized and non-embolized cases are essential to validate these observations and guide evidence-based practice.
